# Reconstructing the Iglói system: historical insights and current relevance for distance running

**DOI:** 10.3389/fspor.2025.1708175

**Published:** 2026-01-13

**Authors:** Bence Kelemen, Zsolt Gyimes

**Affiliations:** 1 Department of Sport Science and Physical Education, University of Agder, Kristiansand, Norway; 2 School of Doctoral Studies, Hungarian University of Sports Science, Budapest, Hungary; 3 Department of Athletics, Hungarian University of Sports Science, Budapest, Hungary

**Keywords:** distance running, interval training, lactate shuttle, Mihály Iglói, sports history

## Abstract

**Introduction:**

Historical training methodologies in endurance running provide valuable insights for contemporary sports science. Mihály Iglói's interval-based system, highly influential between the 1950s and 1970s, produced numerous world records yet remains only partially understood. This study reconstructs Iglói's approach through historical sources, contextualizes it within modern theory, and evaluates its relevance to current elite distance running practices.

**Methods:**

A narrative approach was applied, combining Hungarian and English archival sources with a comparative review of contemporary peer-reviewed literature on international-level distance running training. Data synthesis identified continuities, divergences, and adaptable elements of Iglói's methodology.

**Results:**

Iglói advanced Gerschler's intervals into a complex, high volume system with up to 13 interval sessions per week. Training was primarily based on effort-based short repetitions (100–400 m), complemented by race-specific longer repetitions (600–1200 m). Sessions were organized into sets and sub-sets, interspersed with active recoveries and shakedown strides, while particular emphasis was placed on running form. The system was flexible and individualized, adapting to athletes' goals and event demands. Under this framework, athletes achieved 31 world records and numerous national titles.

**Discussion:**

Contemporary distance running training again heavily focuses on controlled longer and shorter aerobic intervals for improving lactate threshold speed (vLT2). From Iglói's system, re-adaptable elements include race speed–specific but controlled short intervals for aerobic conditioning, combined with harder repetitions, alongside the use of active recoveries and “flush” or recovery sets to enhance lactate shuttle mechanisms. Equally important is the emphasis on economic running form at different parts of the race, supporting both efficiency and tactical readiness.

## Background

What is the value of examining historical training methodologies within sports science? The study of training methodologies has been a central theme in sports science, particularly within the domain of endurance performance (1–5). Early physiological constructs, such as maximal oxygen uptake (VO2max) introduced by Hill in the 1920s, provided a foundation for understanding the limits of aerobic capacity during strenuous exercise (6, 7). Building upon these insights, Gerschler and Reindell formalized interval training in the mid-20th century, emphasizing cardiovascular adaptations and pulse-rate recovery (8–10). By the 1960s, additional constructs, including the lactate threshold, maximal lactate steady state, and ventilatory thresholds (VT1 and VT2), expanded the analytical framework for evaluating endurance performance (11–14). Later, Véronique Billat and colleagues demonstrated the superior efficacy of short, high-intensity intervals near VO2max compared with steady-state training (15), while subsequent high-intensity interval training (HIIT) protocols, such as Tabata's 20/10 model and sprint interval training (SIT), highlighted time-efficient strategies for improving both aerobic and anaerobic capacities (16, 17). More recently, intensity distribution models (Polarized, Pyramidal, Threshold), zone-based training frameworks (3 and 5 zone models), and submaximal endurance modalities (e.g., Zone 2 training) have shaped the contemporary research agenda (18–22).

Despite these advances, limitations persist in the existing literature (23). Much of the experimental evidence is derived from non-elite athletes, often recreational runners or university students, with small sample sizes and short intervention periods (typically 4–6 weeks) (24, 25). Furthermore, many studies focus on isolated training modalities, such as HIIT or continuous Zone 2 training, rather than reflecting the multifactorial and integrative nature of real-world endurance training (26, 27). As a result, findings frequently diverge from coaching practice, where methodological progress often emerges from the interplay of empirical field observations and retrospective scientific validation. Coaches frequently pioneer new approaches based on daily experimentation with athletes, while sports scientists subsequently explain the underlying physiological mechanisms, including metabolic pathways, neuromuscular adaptations, and bioenergetic efficiencies (28). Importantly, modern frameworks often represent refinements of earlier methods, reinterpreted through contemporary science to improve precision, integration, and adaptability. A prominent example is the “Norwegian Double Threshold” method, which was developed in the early 2000s and became internationally adopted and widely used in the 2020s. It was primarily developed and experimented with by the Norwegian long-distance runner Marius Bakken, building upon the integration of Arthur Lydiard's aerobic base training principles (with Per Halle, as coach), Kenyan training practices incorporating lactate threshold measurements (with Frank Evertsen, as scientific advisor), and clustering strategies derived from sprint coaches (Leif Olaf Alves, through collaborative interaction) (29–31).

Within this broader historical trajectory, one major gap remains, the limited scholarly attention devoted to Mihály Iglói, the Hungarian coach whose innovative interval-based training system produced world-class performances and multiple world records in the 1950s (32). Despite his profound influence, accounts of Iglói's methodology are often fragmented, inconsistent, or inaccurate (33, 34). The present study seeks to address this gap by reconstructing Iglói's training system through contemporaneous sources and professional testimonies, situating it within its historical context, and examining its principles from a modern perspective. Furthermore, the study compares Iglói's methods with the training practices of contemporary distance runners and identifies elements that may be reinterpreted and integrated into current elite-level endurance training.

The article is structured as follows. First, the historical context of Iglói's career, life, and personality is presented. Second, his innovative interval training system is reconstructed and described in detail. Third, the analysis examines his methods from a contemporary perspective, identifying elements that can be reinterpreted in light of current knowledge and integrated into modern training paradigms.

## Material and methods

This study employed a narrative research approach, as this format was considered most appropriate for addressing the research question (35). The methodological framework combined two complementary components: a historical document analysis and a qualitative comparative analytical review.

In the first phase, a historical reconstruction was undertaken to examine Mihály Iglói's life, personality, and coaching methodology within their broader historical and cultural context. Sources were analyzed in both Hungarian and English. In addition to peer-reviewed articles, original training manuals, coaching notes, books, grey literature, and selected interview excerpts, discussions were also conducted with athletes who had trained directly under Iglói. These oral-history interviews provided first-hand perspectives on his methods and personality. This multi-source strategy is consistent with the principles of historical document analysis, enabling a comprehensive and nuanced understanding of Iglói's practices and innovations (36, 37).

In the second phase, a comparative analysis was conducted to evaluate Iglói's methods from a contemporary perspective (38). This part relied primarily on the author's previous and ongoing narrative reviews of distance-running training methodologies (39–41). To this end, evidence was extracted from contemporary peer-reviewed literature on middle- and long-distance training practices. The literature search was conducted across multiple electronic databases, including PubMed, Scopus, Web of Science, SportDiscus, and ResearchGate, without language restrictions. Title, abstract, and keywords fields were searched using Boolean operators (AND, OR) with the following key terms: “training”, “distance running”, and “elite-level”. Eligible documents included review articles, original research papers, and case studies.

The extracted information was synthesized to identify continuities, divergences, and potentially transposable elements of Iglói's methodology that may be reinterpreted in light of current knowledge and integrated into modern endurance training paradigms.

## Results

### Historical background

The origins of modern year-round distance running are attributed to Finnish coach Lauri Pikhala in the early 20th century, whose athletes included Paavo Nurmi and Hannes Kolehmainen (42). Winter training emphasized long endurance runs and hiking, while in preparation for the summer season, early forms of interval-like training were introduced, combining sustained race-pace efforts with faster segments and short sprints (1, 10). In parallel, Swedish coaches developed the “natural” fartlek, integrating variable speeds over hilly forest terrain across 5–12 km (43, 44). These approaches laid the groundwork for subsequent world-record performances, with athletes such as Gunder Hägg and Arne Andersson approaching the 4-min mile and Hägg breaking 14 min over 5,000 m (45).

The true breakthrough in endurance training emerged in the late 1930s with German coach Woldemar Gerschler, who, in collaboration with cardiologist Herbert Reindell, developed a scientifically grounded system later known as the Freiburg Interval Training (46). In experiments involving 3,000 subjects over 21 days, Gerschler and Reindell observed significant increases in heart volume and performance, leading to the design of short, fast repetitions interspersed with recovery periods, termed “intervals”, during which the primary training effect occurred (9). Athletes performed repetitions of 100–200 m at high intensity (later up to 400 m), resuming the next repetition only after heart rate declined to approximately 120 bpm, typically within 90 s (47). The Freiburg Interval Training approach also incorporated additional “supramaximal runs” of 400–2,000 m at race pace with full recovery, emphasizing anaerobic capacity development (48). The method produced rapid and substantial improvements in performance, as demonstrated by athletes such as Rudolf Harbig, Josy Barthel, and Roger Moens. Beyond its rigor and monotony, this system highlighted that the primary adaptation occurs during recovery intervals rather than during the repetitions themselves, forming a cornerstone of contemporary endurance coaching (49).

Building on this success, by the 1950s, interval training had become widespread, exemplified by Emil Zátopek's unparalleled triple Olympic victory in 1952, achieved through very high volumes of 200–400 m repetitions (10). His daily sessions often included sets such as 5 × 200 m, 40–50 × 400 m, and an additional 5 × 200 m, each repetition interspersed with 200 m of active jogging, though primarily performed at submaximal intensity (49). Coaches such as Bertl Sumser and Franz Stampfl, the latter credited with coaching Roger Bannister to the first sub-four-minute mile, further refined the interval approach by introducing longer repetitions of 800–2,400 m with equal-distance active recoveries (50, 51). Stampfl also implemented systematic seasonal progression, gradually increasing interval intensity month by month. At that time, it was not uncommon for athletes to train with intervals five to seven days per week (see Table 1) (49).

**Table 1:** Example week from Franz Stampfl's training plan for an 800 m runner at different times of the year [source (52)].

Day	November	May
Monday	6x 440 yards in 66 s (440 yard jog in 3 min)+ 4 × 150 yard in 18 s (3–5 min rest)	8 × 440 yard in 57 s (440 yard slow jog)
Tuesday	3x 880 yard in 2:15 (880 yard jog) + 6 × 150 yard sprints	6x 660 yard in 1:28 (660 yard slow jog)
Wednesday	10 x220 yard in 29–30 s (220 yard jog)	10x 330 yard in 41 s (330 yard slow jog)
Thursday	6x 440 yards in 66 s (440 yard jog in 3 min)+ 4 × 150 yard in 18 s (3–5 min rest)	20x 100 yard from starting block in 11.5 s (3 min walk back rest)
Friday	6–8 miles fartlek	8 × 440 yard in 59 s (440 yard jog)
Saturday	Calisthenics	Fartlek
Sunday	Rest	Rest

1 yard = 0.914 m; 1 mile = 1,609 m.

### Life and work of Iglói

Mihály Iglói, originally born as Ignácz Mihály on September 5, 1908, in Eger, Hungary, experimented with multiple sports in his youth, initially practicing gymnastics and later competing in pole vault (53). Upon moving to Budapest, he increasingly focused on running, enrolling in the Hungarian University of Physical Education in 1929. He drew inspiration from the legendary Finnish runner Paavo Nurmi, as well as the Polish Olympic champion Janusz Kusociński, whose pre-Gerschler preparation already incorporated intervals, including 15× 200 m repetitions, in his training for the 10,000 m gold at the 1932 Olympics (32).

Iglói graduated in 1934 and began working as a physical education teacher while competing for the BBTE. In 1936, he represented Hungary in the 1,500 m at the Berlin Olympics but did not progress beyond the heats. In this period, reflecting the era's nationalistic tendencies, he Magyarized his surname from Ignácz to Iglói (54). He traveled twice to Finland and also trained in Sweden and Germany, gaining firsthand exposure to elite athletes and directly studying their training methodologies (55). His visit in Germany likely exposed him to the Freiburg Interval System, which may have influenced the development of his own approach (32). In 1938, at the Hungarian–Finnish match, he defeated the 1932 Olympic champion Lauri Lehtinen and medalist Lasse Virtanen over 3,000 m. Even at this stage, he began experimenting with his own training methods. In 1939, he ran a world-record time as part of a 4 × 1,500 m relay team, though the record was not ratified due to wartime conditions. His athletic career was interrupted by World War II, during which he served in the military and subsequently spent five years in Soviet captivity (54).

After the war, he returned to Hungary, pursued historical studies, and obtained a university professorship. From 1950, he worked as a physical education teacher at Árpád Gymnasium in Óbuda. During the Soviet-inspired reorganization of Hungarian sport, he joined Budapesti Honvéd in 1951, where the “sports company” was established (53). Here, he began coaching talented runners such as Sándor Iharos and István Rózsavölgyi, who by 1953, alongside Garai and Béres, set a world record in the 4 × 1,500 m relay. Later, László Tábori joined, forming the legendary “Hungarian trio”. Occasionally, other runners such as Ferenc Mikes, Lajos Kovács, and Béla Szekeres supplemented the group, also achieving relay records (56). The true breakthrough came in 1954, with 1955 representing the pinnacle of Hungarian middle-distance running. Over two years, his three leading athletes set a total of 21 world records and 25 European records. In 1954, Hungarian runners occupied the top three positions in the global rankings for 1,500 m. By the end of 1955, Hungarian athletes held all world records between 1,000 and 10,000 m. Sándor Iharos's performance was particularly remarkable: he broke seven world records individually and was named “European Sportsman of the Year” in 1955, remaining the only Hungarian athlete to receive this honor (127).

The 1956 Hungarian Revolution and subsequent Soviet occupation abruptly ended this success. Iharos did not attend the Olympics, Rózsavölgyi performed below form, and Tábori finished fourth and sixth in the finals (56). Due to political uncertainty and Iglói's previous Soviet captivity, he and Tábori decided to emigrate, settling in the United States. From 1956 to 1957, they participated in an American competitive tour organized by Sports Illustrated, and after two years of uncertainty (57), Iglói first coached at the Santa Clara Valley Youth Village. He later founded the Los Angeles Track Club, later his next team was the Santa Monica Athletic Association (renamed the Santa Monica Track Club in the fall of 1972 under Joe Douglas) (58). Through his coaching, previously internationally modest U.S. middle-distance running produced world-class athletes (59).

Jim Beatty rapidly improved under his guidance, setting American records in the mile and 5,000 m in 1961, and in 1962 became the first runner to complete an indoor mile in under four minutes. Max Truex joined in 1959, finishing sixth with a new American record in the 10,000 m at the 1960 Rome Olympics. In 1961, additional outstanding athletes such as Jim Grelle and Bob Schul joined. Schul trained independently from 1963 but using Iglói's principles, winning Olympic gold in the 5,000 m in 1964 with a final 300 m sprint of 37.8 s (60).

In the early 1960s, American runners successively broke records: Beatty lowered the U.S. 5,000 m record to 13:55 in 1963, Truex further advanced U.S. distance running with 13:49, and Grelle reached the world elite in the mile with a national record of 3:55.4. 1964 marked a pinnacle for U.S. distance running, yet many of his protégés retired (Beatty and Tábori), while the rising influence of Arthur Lydiard's training methods from New Zealand overshadowed Iglói's approach. This was partly due to his secretive style: during his Hungary period, he encoded training plans for central oversight and applied extensive individualization, adjusting workouts daily, which made his methods difficult to replicate (59).

In 1970, Iglói moved to Greece, continuing his coaching career with the national team. His athletes established 157 national records, including Michalis Kousis, who ran 2:06 in Split for the marathon, though it was not recognized as a world record due to the track being 900 m short (61). In 1993, he returned to Hungary and lived in Budapest until his death in 1998 at the age of 90 (59, 62).

Iglói was renowned for his extraordinary discipline, systematization, and autocratic, uncompromising leadership style (55). Athletes had no input into the training plans. Due to his strict and disciplined coaching style, combined with his original surname (Ignácz), his athletes themselves coined the nickname “Uncle Nazi.” This informal designation reflected both linguistic association and perceived rigor, while his consistency, exemplary conduct, and exceptional motivational capacity were widely regarded as key factors in maximizing athletes' performance and work ethic (63). Numerous anecdotes attest to his dedication; for example, he would sit on snow in subzero temperatures until his athletes completed training, demonstrating his determination (56). The athletes under his guidance collectively achieved remarkable success across multiple levels: 49 Hungarian national records, 2 Balkan records, 45 U.S. national records, 25 European records, 157 Greek national records, and 31 world records. Beyond these achievements, they secured numerous championship titles, including 41 Hungarian national championships, 27 U.S. national championships, 2 U.S. collegiate championships, 1 British championship, and 35 Greek national championships (62). His most successful athletes are summarized in Table 2**.**

**Table 2:** Most successful athletes of Iglói [source (64, 65)].

Name	Personal Bests	Achievements
Sándor Iharos	1,500 m: 3:40.8 2,000m: 5:09.8 3,000 m: 7:55.6 5,000 m: 13:40.6 10.000 m: 28:42.8	12 World Records (8 Individual) 31 Times Hungarian Champion 1,955 Sportsman of the Year by Helms Athletics Institution
István Rózsavölgyi	1,000 m: 2:19.0 1,500 m: 3:38.8 1 mile: 3:59.0 2,000m: 5:02.0 3,000 m: 7:52.4 5,000 m: 13:55.4	7 World Records (Individual) 19 National Records 1,960 Rome Olympics: 3rd place in 1,500 m
László Tábori	1,500 m: 3:40.8 1 mile: 3:59.0 2,000m: 5:03.0 3,000 m: 7:58.0 5,000 m: 13:50.8 800 m: 1:50.8	3 World Records (1 Individual) 4th place 1,956 Olympics 1,500 m 3rd man to run a sub 4 min mile
Robert Schul	1,500 m: 3:40.7 1 mile: 3:58.9 2 miles: 8:26.4 3,000 m: 7:59.0 5,000 m: 13:38.0	1,964 Tokyo Olympics Champion in 5,000 m World Record over 2 mile National Record over 2 mile and 5,000 m
James Beatty	1,500 m: 3:39.4 1 mile: 3:55.5 2 miles: 8:29.8 5,000 m: 13:45.0	First Indoor Sub 4 Minute Mile 11 National Records 3 World Records
James Grelle	1,500 m: 3:38.5 1 Mile: 3:55.4 2 Miles: 8:32.5 5,000 m: 14:10.8	American Record Holder over the Indoor and Outdoor Mile and 2 Miles
Max Truex	5,000 m: 13:49.6 10.000 m: 28:50.34	6th in 10,000 Olympics in 1,960 National Record Holder over 10.000 m
Michalis Kousis	5,000 m: 13:35.4 10,000 m: 28:44.4 Marathon: 2:14.36	National and Balkan Records 4th at 1,980 Boston Marathon 3-time Olympian (1,978, 1980, 1984) 1979 Mediterranean Games Winner

### The Iglói training system

The training system developed by Mihály Iglói evolved continuously throughout his coaching career, both in terms of training volume and complexity, from his early years in Hungary to his later work in the United States, and Greece (63). Because Iglói never formally published his methods, and often kept them deliberately coded or secret, information about his system has frequently appeared fragmented or even contradictory (32, 33, 59). Contemporary accounts sometimes relied on rival coaches who sent “observers” to his sessions, producing reports of questionable accuracy, particularly regarding split times and session structure (Covbey, 2011; 49).

Despite these inconsistencies, several core principles remained stable throughout his career. Training frequency was almost always thirteen sessions per week, meaning two sessions daily except on Sundays for his high level runners. The majority of these sessions took place on a 400 m cinder track or the infield grass, and almost exclusively consisted of interval training performed in racing spikes (66). Long continuous runs outside the track were rare, although occasional steady runs were held in parks on Fridays or Sundays during fall and spring. This highly controlled approach stemmed not only from Iglói's methodological preferences but also from his lack of trust in athletes' compliance, since even world-record holders under his guidance were known to skip prescribed training loads if left unsupervised (49, 67, 68).

Although initially inspired by the Freiburg Interval System, the Iglói system grew substantially more complex over time (32, 69). A hallmark of his coaching was the strong emphasis on individualization: sessions were tailored to the needs of each athlete, rarely repeated in identical form, and frequently adjusted on the spot depending on the runner's physical state, movement quality (32, 70, 71). Iglói himself stated that he did not train according to a workout pattern made long in advance, planning instead from day to day based on how the athlete felt, environmental conditions, and training objectives (72). Athletes were typically organized into groups of five of similar ability, occasionally supplemented with weaker runners to alter group dynamics (66).

### Workout structure and components

Training sessions were composed of sets (usually 3–6 total sets) of intervals interspersed with active recovery. Recoveries were usually performed at half the distance of the preceding repetition, such as a 50 meter jog after 100 meters, while longer breaks between sets involved 400 to 800 m of jogging (68). As a result, sessions often lasted several hours, with athletes rarely stopping and heart rates generally remaining above 120 beats per minute throughout (66). Intervals were always grouped into sets, with athletes aware only of the current set, not the overall session structure. Within and between sets, pace/effort changes were frequent. Between the main sets, clearance set periods, comprising 8–10 × 100 m at Fresh pace or other light efforts of approximately 150 m, were strategically inserted to sustain training quality while facilitating recovery, keeping the athletes' heart rate elevated (69, 71).

Sessions always began and ended with ten times 100 m at defined efforts, for example alternating between “fresh” and “good” or between “fresh” and “easy.” The first series of 100 m served as a diagnostic tool, enabling Iglói to assess the athlete's condition and adjust the session accordingly, while the final series functioned as a cooldown designed to “flush” the system (66, 73). Occasionally, a tester repetition was incorporated at the end of training, allowing Iglói to assess how fast athletes could run under accumulated fatigue. The main sets represented the most demanding elements of training, while clearance sets were used not as passive recovery but as moderated efforts that maintained physiological stress while enabling athletes to recover sufficiently to perform additional high-quality repetitions (33). This design allowed for an increase in both total training volume and time under tension without overloading athletes to the point of breakdown. Table 3 shows a representative session for László Rózsavölgyi, one of Iglói's top athletes, and illustrates the general outline of the workouts.

**Table 3:** General outline and example of a training session in the Iglói system [source (68, 71)].

General Outline	Workout Example
Warm-up	10 × 100 m in 20 s (50 m jog recovery)
Main set 1.	10 × 300 m in 45–48 s (100 m jog recovery)
Clearance period	10 × 100 m (50 m jog recovery)
Main set 2.	5 × 600 m in 1:40 (200 m jog recovery)
Clearance period	10 × 100 m (50 m jog recovery)
Main set 3.	10 × 300 m (100 m recovery)
Clearance period	-
Optional Test rep/set	-
Cool-down	10 × 100 m in 15 s (50 m jog recovery)

In his early Hungarian period, Iglói's conditioning intervals mostly consisted of 100–400 m repetitions (69, 73). Later, especially during his American period, he extended intervals to 400–800 m for longer-distance runners (66). Pre-competition training incorporated harder efforts at or beyond race pace, usually in small numbers of one or two repetitions, ranging between 400 and 1,200 m (68). A famous example was Sándor Iharos's 3,000-m world-record preparation, which included two times 1,200 m in 2:57 and 3:01 with five minutes recovery. Another tradition within the group was to run 1,200 m hard followed by an all-out 400 m on New Year's Day (32). Iglói also employed timed runs and maximal repetitions as psychological and diagnostic tools, both to test fitness and to reduce athletes' anxiety about running fast. As his career progressed, such timed runs were introduced earlier in the season (63).

Daily training was divided into two sessions. The morning workout was not merely a warm-up but often represented one-third of the total daily load. It typically lasted around one hour and consisted primarily of light intervals (60 and 100 m), or sets such as: two times 100 m, two times 200 m, two times 300 m, two times 100 m, and two times 60 m. Early repetitions were relatively slow, for example 100 m in 20 s, but gradually increased in speed as the body warmed up (56, 73). The afternoon session was longer and more intensive, often lasting several hours (74). Overall weekly mileage under Iglói ranged from 60 to 100 miles, that is 100 to 160 km (or even more for marathoners), depending on the athlete's event specialization and level. In his American period, some of the hardest interval sessions extended to three or even four hours in duration, reflecting the extremely high training volume (67, 75). Table 4 presents a representative 10 day period from István Rózsavölgyi during the competition season.

**Table 4:** Excerpt from István Rózsavölgyi's training log between July 18 and July 28, 1955 [source (56)].

Day	AM:	PM:
Monday	2,000m easy 30 × 100 m easy	3,000 m easy 15 × 100 m 3x(4 × 200 m) 3x (10 × 100 m fast buildup)
Tuesday	2,000m easy In 15 laps: 2 × 60 m very hard, 2 × 100 m steady	3,000 m easy 15 × 100 m easy 3x (3 × 300 m in 45s, with 100 m jog) 6 × 100 m 2x 400 m (49,6;52,8 s) with 800 m easy jog
Wednesday	2,000m easy In 15 laps: 2 × 100 m fast, 2 × 100 m easy	3,000 m easy 2x (10 × 100 m easy, fast) 4 × 200 m in 29 s, with 50 m jog 3x (4 × 150 m fresh)
Thursday	2,000m easy In 15 laps: 2 × 60 m very hard, 2 × 100 m easy, 2 × 100 m very hard	3,000 m easy 15 × 100 m easy 600 m-1:18,6 3x (10 × 100 m easy)
Friday	2,000m easy In 15 laps: 2 × 100 m fast, 2 × 100 m easy	50 min easy
Saturday	2,000m easy In 15 laps: 2 × 60 m very hard, 2 × 100 m fast	3,000 m easy 15x 100 fresh 3x (2 × 200 m in 30 s, 2 × 200 m in 27 s)
Sunday	2,000m easy In 15 laps: 2 × 100 m good, 2 × 100 m fresh	3,000 m easy 10 × 100 m easy 2 × 100 m fast 10 × 100 m fresh
Monday	30 min easy	3,000 m easy 3x (4 × 100 m fresh) 4 × 150 m hard
Tuesday	Travel to Helsinki	3,000 m easy 12 × 100 m easy 6 × 150 m 3 × 250 m fresh 5 × 100 m
Wednesday	2,000m easy 10 × 100 m easy 4 × 150 m fresh	3,000 m easy 6 × 100 m easy 4 × 100 m good 4 × 100 m steady **800 m race (2.), 1:49.6 (400 m split: 56 s)**
Thursday	2,000m easy 12 × 100 m easy 4 × 150 m fresh	3,000 m easy 8 × 100 m easy 4 × 100 m good 4 × 100 m steady **1,500 m (2.), 3:42.8 (56.6–1:55.7–2:56.7)**

Competitions indicated in bold.

### Periodization: annual and weekly cycles

Most elements of Iglói's training plan were present throughout the year, making it difficult to draw sharp distinctions between cross-country and track phases. The yearly training cycle generally began in late August and lasted until early August of the following year, when athletes were granted a two-week rest (66). In his early Hungarian period, Iglói structured the annual cycle into three broad phases (see Table 5), gradually increasing the length and intensity of the intervals as well as the number of completed sets (73).

**Table 5:** Annual distribution of Iglói's periodization during the Hungarian period [source (73)].

Period	Repetition Distance	Repetitions per Set	Number of Sets	Intensity/Purpose
Period I: Main period	100–400 m	10–20	2–3	Run at racing speed plus the athlete's relative speed
Period II: Training period	50–100 m	5–10	5–6	Performed at relative speed plus lighter efforts, intended to build competitive condition
Period III: Racing period	50–200 m	3–6	3–4	Run at racing speed plus the athlete's relative speed

Autum and early spring months acted as the base phases of Iglói's program, while the late spring and summer track meets served as the primary competitive goals (56). This heavy reliance on track-based training contributed to his athletes' consistent indoor-season success, since the fall and winter already involved enough high-quality running to maintain sharpness. Iglói did not aim to bring athletes into shape too quickly, instead, he believed that when an athlete was ready, he was simply ready, and there was no reason not to stay in competitive form. High-quality training and racing were present across all phases of the year, and the program fostered a high degree of lactate tolerance. Specificity in training was carried to the extreme, with virtually all but the easiest runs performed in racing spikes, sometimes covering more than 16 miles in a single session (66, 75, 76). The training year extended nearly twelve months, with only two scheduled breaks: Christmas Day and the two-week pause in late August.

The weekly cycle was designed with variation in load, balancing harder and easier days while maintaining a consistently high overall volume. Three days: Wednesday, Friday, and Sunday were usually considered “easy days,” with lighter morning and afternoon sessions. These afternoons often included 100 meter strides of varying speeds, known as “shakedowns.” Occasionally these were extended to 200 m, serving both as recovery and maintenance of technical rhythm. Iglói frequently emphasized the variety of his program, once remarking that he had devised one hundred thousand different workouts (66).

During the American days Monday was typically the longest and most demanding day, with morning sessions covering up to nine miles and afternoon sessions sometimes extending to twenty-three miles. Workouts often featured large volumes of untimed intervals, for example 40 × 200 m at varying speeds with 100 m recovery. Tuesday reduced the volume slightly, focusing on sets of 400, 600, or 800 m repetitions, with the pace of these intervals sharpening as the season progressed, not according to a rigid timetable but in line with the athlete's individual development. Wednesday, Friday, and Sunday remained lighter, dominated by easy running and shakedowns, while Thursday provided a moderate workload, with intervals at differing speeds over varying distances. Repetition work, such as sets of 400 and 600 m was sometimes introduced on Thursdays at controlled paces. Saturday was again hard, often the most intense day of the week, featuring sets of 600 or 800 m interspersed with shorter repetitions at different speeds. It was not uncommon for these sessions to consist of either 12–20 × 400 m in sets, sometimes finishing with an all-out 400 meters after a demanding series of longer repetitions, or, in the U.S., 30–60 × 200 m in various sets, often with 100 meters of shakeouts in between (66, 76).

This weekly rhythm reflects both structure and flexibility: while general patterns existed, the actual design of sessions remained highly individualized and subject to daily adjustments. The result was a training system that, while extremely demanding, continually adapted to the condition of the athletes and maintained a balance between volume, intensity, and recovery. Tables 6, 7 present a sample week from different phases of the American training period.

**Table 6:** Training week from the American period—pre-competition period [source (66)].

Period	Monday	Tuesday	Wednesday	Thursday	Friday	Saturday	Sunday
**AM:.**	**1,200 m run; 2x (8 × 200 m hard speed/ 100 m; 8 × 200 m ggod swing/ 100 m) 300 m between sets; 6 × 150 m all-out buildup**	1,200 m run; 2x (8 × 200 m good swing/ 100 m; 8 × 200 m hard speed/ 100 m) 600 m between sets	*6 miles 6* *×* *200 m good speed during run; 6* *×* *100 m shakedown*	1,200 m run; 10 × 200 m good speed/ 100 m; 300 m; 10 × 200 m good swing/ 100 m; 300 m; 10 × 200 m hard speed/ 100 m; 4 × 100 m shakedown	*15 laps on large field, every 3rd lap good swing; 15x 100 m shakedown.*	**1,200 m run; 20 × 200 m good speed/ 100 m; 400 m jog; 10x (2 × 150 m very hard speed/ 50 m) 150 m between sets.**	**4 miles; 15 × 100 m shakeup; 1 × 800 m (2:04); 600 m jog; 3x 800 m (2:12)/ 400 m; 600 m jog; 1 × 800 m (2:08); 2 miles jog; 9 × 200 m very hard speed, every 3rd all out/ 200 m; 10 × 100 m shakedown.**
**PM:.**	2 miles; 15 × 100 m shakeup; 10 × 300 m good speed/100 m; 800 m jog; 5x(6 × 250 m/150 m) 1 set good swing, 3 sets hard swing, 1 set good swing, 400 m between sets; 1,200 m jog; 20 × 200 m very hard speed, every 4th all out build-up/ 200 m; 20 × 100 m shakedown	2 miles; 15 × 100 m shakeup; 10x (4 × 200 m good speed/ 100 m), 200 m between sets; 1,200 m jog; 9 × 600 m/300 m (3 good swing, 3 hard swing, 3 good swing), 1,200 m jog, 12 × 200 m very hard speed/ 200 m; 4 × 100 m shakedown	*6 miles; 15* *×* *100 m shakeup; 10* *×* *150 m/50 m alternate 1 good speed build-up and 1 good swing; 10* *×* *100 m shakedown.*	**2 miles; 15 × 100 m shakeup; 10 × 150 m good speed/50 m; 400 m jog; 2 × 600 m (1:33)/ 300 m; 600 m jog; 10 × 150 m good swing/50 m; 400 m jog; 2 × 600 m (1:33)/300 m; 600 m jog; 10 × 150 m/fresh swing/50 m; 400 m jog; 2 × 600 m/300 m, first at 1:33, second all out; 2 miles jog; 15 × 100 m, 1 good speed build-up, 2 very hard speed; 10 × 100 m shakedown**	*6 miles with a few 150 m good speed build-up; 16* *×* *150 m/50 m alternate 1 good speed build-up and 1 good swing*	2 miles; 15x 100 m shakeup; 10x (3 × 200 m/100 m hard spedd), 300 m between sets; 1,200 m jog; 16 × 250 m/ 150 m, good swing; 600 m jog; 1 × 400 m all out; 20 × 100 m shakedown	

Bold indicates hard training days, regular font indicates moderate training days, and *italic* indicates low-intensity training days.

**Table 7:** Training week from the American period—competition week [source (66)].

Period	Monday	Tuesday	Wednesday	Thursday	Friday	Saturday	Sunday
**AM:.**	15 laps around large field, every 2nd lap 1 × 300 m good swing, every 3rd lap 2 × 150 m good speed build-up	*7 miles in park*	*7 miles in park*	*One hour in park*	*6 miles; 20x 100 m shakedow, with a few good speed build-up*	*40 min easy; 20x 100 m shakedown, with a few good speed build-up*	*20 min; 3x (8* *×* *150 m good speed/50 m); 300 m between sets*
**PM:.**	**4 miles; 15** **×** **100 m shaeup; 6** **×** **100 m good speed build-up alternated with good swing build-up; 2** **×** **150 m good swing/ 50 m; a few wind sprints; 1** **×** **1,200 m (3.05); 1,200 m jog; 10** **×** **150 m fresh swing/ 50 m; 1** **×** **400 m all out; 600 m jog; 20** **×** **100 m shakedown**	**2 miles; 15x 100 m shakeup; 10** **×** **150 m good speed/ 50 m; 600 m jog; 10x 250 m good swing/ 150 m; 1,200 m jog; 4** **×** **400 m (59–60 s), 200 m between; 1,200 m jog; 10x 150 m good speed/50 m; 600 m jog; 10x 250 m good swing/ 150 m; 1,200 m jog; 3x 250 m very hard speed/250 m; 20** **×** **100 m shakedown**	*8 miles a few 200 m at good swing; 20* *×* *100 m shakedown*	*8 miles a few 200 m at good swing; 20x 100 m shakedown*	*40 min jog; 20* *×* *100 m shakedown with a few good speed build-up*	*30 min jog; 10x 100 m shakeup; 6* *×* *100 m good speed build-up, every 2nd 1* *×* *150 m good swing,* **5,000 m race**	

Bold indicates hard training days, regular font indicates moderate training days, and *italic* indicates low-intensity training days.

### Interval distances, recoveries, and effort system

Iglói's training made use of a wide variety of interval distances, generally between 100 and 1,200 m (see Table 8). The shortest runs of 100 m were typically performed after the traditional 2-mile warm-up (shake-up) or at the end of training as part of the cool-down (shake-down). Distances between 125 and 300 m were used most often in a classic interval format, with recoveries about half the length of the repetition. Common sets included 6 × 150 m, 8 × 200 m, or 4 × 300 m. On days with higher volumes, Iglói frequently used “sandwich patterns,” such as 6 × 150 m, 4 × 300 m, and another 6 × 150 m. Sequences like these could be repeated several times depending on the event or the runner's ability. Middle-distance runners focused more on shorter, faster intervals, while 5,000 m runners typically completed larger sets with longer but less intense repetitions. The following table summarizes the typical recovery distances prescribed between repetitions (see Table 8) (66, 77).

**Table 8:** The interval length and recoveries in the Iglói method [source (66)].

Interval Distance	Recovery Distance
100–150 m	50 m jog
200 m	100 m jog
250 m	150 m jog
400 m	200 m jog
600 m	300 m jog
800–1,200 m	400–1,200 m jog

Shorter repetitions of 125 m and 175 m were considered speed work and always followed by a full jog of equal distance. Between sets, recovery runs typically ranged from 400 to 1,200 m. Consequently, training blocks within the Iglói system were characterized by continuous movement, with no complete stops or walking breaks. The only occasional interruption occurred prior to longer or more demanding sets, when athletes briefly approached the coach to clarify the structure of the upcoming block; during these recovery runs, jogging pace was generally maintained at approximately 5 min/km. After demanding sessions, athletes changed into training shoes and continued with shake-downs, rather than finishing abruptly. Most interval work itself was done in spikes, with only warm-ups, cool-downs, and easy days in training shoes (66).

Iglói's system emphasized effort-based intensity rather than strict times, allowing athletes to progress naturally throughout the season. The vast majority of the intervals were performed at relatively moderate intensities, run in a relaxed manner at “fresh” and “good” speeds, and thus were most likely predominantly aerobic in nature (78). Most athletes were not aware of exact split times, and the majority of repetitions were not formally timed during regular sessions. A “fresh” 100 m repetition approximately corresponded to 5 km pace, whereas a “good” effort aligned more closely with 1,500 m or mile race pace/effort (75, 76). For longer repetitions, an approximate illustration can be provided using the example of an athlete with a 4:15 mile personal best (∼64 s per 400 m pace as 100% of race pace): *fresh* ≈ 90 s (∼70% of race pace), *good* ≈ 70 s (∼90%), and *hard* ≈ 60 s (∼105%) (77). This meant that at the beginning of the season, a “good” 200 m might take an athlete around 30 s, whereas by the end of the season it could drop to 26–27 s (75, 76, 79). The main effort levels were as follows (see Table 9). It should be emphasized, however, that the Iglói system was fundamentally effort-based rather than strictly intensity-prescribed. The relative intensity associated with each effort category varied across repetition lengths, training phases, and the athlete's momentary condition. Accordingly, the percentage values presented here, both in terms of % of race pace and % of maximum heart rate, serve solely as illustrative approximations rather than fixed physiological targets.

**Table 9:** The effort system in the Iglói method [source (66, 79)].

Effort Level	% of Max HR	Description
Fresh	∼60%	Relaxed, long stride (*swing tempo*), little or no shoulder tension. Used early in the season or for steady aerobic strength runs and shake-outs
Good	∼70%	Most common intensity. Shoulders slightly tense, rest of the body relaxed. Performed at both *swing* and *speed* tempos
Good Build-Up	∼70%–80%	Starts at Good pace and gradually builds faster depending on session goal
Hard	∼80%	7/8 speed, under control. Frequently used by 800–1,500 m runners once adequate conditioning was reached
Very Hard	∼90%	Fast leg turnover, strong arm action, high knee lift. Used as speed work at the end of practice
All-Out	100%	Maximal effort, typically at the end of a session. Focus on visible strain rather than perfect form

The first three effort levels (“Fresh, Good, and Hard”) each incorporated two forms of running technique. During his American period, Iglói developed these two distinct styles of running. The swing tempo featured an elongated stride with a lower knee drive and was primarily used during the early stages of a race, either for pacing or following. In contrast, the speed tempo involved a shorter, quicker stride with a high knee lift and active heel recovery, and it was applied during surges or finishing kicks to maximize speed and efficiency (66, 76).

### Psychological and adaptive elements

The Iglói system also created a distinctive psychological environment. Training was entirely coach-directed, with minimal athlete input or analysis. Athletes were not expected to understand the logic of the sessions, but rather to place absolute trust in the coach. This required a high level of motivation and in some cases relied on the use of fear as a motivating factor. Importantly, the system also used hard repetitions and timed efforts to teach athletes to cope with the fear of fatigue and the fear of racing itself, thereby preparing them both physically and mentally for competition (56, 57, 74).

### Afterlife

The Iglói approach continued to exert influence well beyond his own career, as his former athletes became coaches themselves and achieved international success building upon his methods. Over time, however, they adapted the system based on new knowledge and personal observations, while maintaining its cornerstone principle of high-volume, short, fast, but predominantly relaxed, effort-based intervals.

Notably, following the events of 1956, István Rózsavölgyi, who remained in Hungary and trained independently, secured a bronze medal in the 1,500 m at the 1960 Rome Olympics, and his 3:38.6 Hungarian championship record remains unbroken six decades later (56). Subsequently, Bob Schul, returning to Ohio in the autumn of 1963, prepared alone through the harsh winter for his 1964 Olympic 5,000 m victory (60). Training under challenging conditions, without adequate facilities he initially used three grass hockey fields for straight 300 m repetitions and, when snow covered the ground, relied on an old, unbanked indoor track beneath a football stand. Schul ran very high volumes of fresh, short 100–200 m intervals, using passive walking or walk-jog recoveries instead of continuous jogging; one of his staple weekly harder sessions was 20 × 400 m per week (79–81).

During his California years, László Tábori also coached using Iglói's principles, later supplementing the shorter intervals with longer, relaxed 1,200–2,400 m repetitions, which, from a modern perspective, can be considered lactate-threshold intervals. He also used longer, harder intervals in a “ladder” format, that is, with progressively shorter distances in each repetition (77). Joe Douglas and Merle McGee, a successful coach at the Santa Monica Track Club in the 1970s and 1980s, trained numerous 800 m runners who achieved 1:42–1:43-level international performances, including Johnny Gray, who later became a coach himself and guided athletes such as Duane Solomon and Khadevis Robinson to similar elite 800 m results in the 2010s (82, 83). Another American coach, Norman Higgins, also applied the methodology, guiding successful female athletes such as Janice Merrill to U.S. records over 1,500–3,000 m. Higgins maintained the classic Iglói pattern of two daily interval workouts on Monday and Tuesday, integrating longer, easier runs and a more pronounced hard–easy training pattern (84).

In Hungary, several coaches adopted elements of Iglói's philosophy, including János Török, one of the last athletes to train directly with Iglói between 1978 and 1980 (63). His athletes achieved national records in the 1,500 m, mile and the 3,000 m during the 1980s (54), some of which remain unbeaten to this day.

## Discussion from a modern perspective

The review key findings are the following: Based on the review of the literature, it can be concluded that Iglói's training method was not as radically different from the prevailing practices of his era as is often assumed, since daily interval training was already dominant at the time, but he introduced unique modifications to the otherwise simple and widely used interval systems, achieving excellent results with his athletes. Building on Gerchler's Freiburg interval system, he mainly used shorter 100–400 m intervals with active recovery, and longer (400–1,200 m) hard anaerobic efforts for pre-race specific conditioning. Contrary to many modern misinterpretations, his program was not merely a form of High-Intensity Interval Training (HIIT), but rather a much more complex, year-round system built primarily on large training volumes and relaxed, effort-based short repetitions, integrating a wide range of speeds and exertions throughout most of the year, including: aerobic intervals, anaerobic repetitions, and speed development, while closely following the annual progression of running paces. One of his most striking innovations was the creative use of different sets and sub-sets, including “shake-up” recovery sub-sets inserted between harder sections. He emphasized the importance of economical running mechanics and the use of varied movement patterns across different tactical phases of competition. Beyond the methodology itself, Iglói's personality as a coach, authoritative, uncompromising, precise, and highly motivational, was crucial in fostering athlete commitment to the large training volumes. Weekly volume (100–160 km, or higher for marathon runners) and training frequency (up to 13 sessions per week) were consistent with modern elite practices (19, 85, 86), but his exclusive reliance on interval training, together with a less obvious yet present modulation of lighter and heavier training days, represented a clear distinction, as he achieved various training goals and physiological adaptations by manipulating the effort, length, volume, and recovery of intervals. In the following, I will discuss in detail the four most adaptable and innovative elements of his system.

### Aerobic intervals

According to Casado et al., the annual training of today's distance runners is largely characterized by high volumes of continuous aerobic running at low intensity. Alongside this foundation, sessions at medium and higher intensities are typically structured as tempo runs or lactate-threshold intervals (between LT1 and LT2), as well as short race-pace repetitions (>800 m) (87). In the final preparation phase, longer and more specific intervals are introduced to better simulate competitive demands (85, 88). Since the 2010s, the so-called Norwegian method has gained increasing prominence (40, 42, 89). Its core principle is the systematic development of the second lactate threshold speed (vLT2), controlled through internal load monitoring such as blood lactate and heart rate measurements, primarily using large volumes of interval training (2). In practice, this often takes the form of twice-weekly “double threshold” days, where athletes perform interval sessions both in the morning and afternoon. Morning sessions typically consist of longer efforts of 6–10 min at LT1 intensities (1.5–2.5 mmol·L^−1^), whereas afternoon sessions emphasize shorter repetitions such as 1,000 m intervals (approximately three minutes) or 25 × 400 m with 30-second recovery at around 10 km pace, both sessions kept below LT2 intensities (2.5–3.5 mmol·L^−1^ for elite distance runners) (2, 90, 91). Marius Bakken, one of the early promoters of the method, even advocates formats such as 25–30 × 45 s with 15-second walking recovery at 5–10 km pace, performed at controlled lactate levels (≤3.5 mmol·L^−1^) (30, 92). These short repetitions at sub-lactate-threshold intensity allow for the consistent accumulation of large training volumes (40–50 km per week of aerobic intervals (19). By maintaining controlled lactate concentrations, such protocols optimize physiological adaptations through enhanced mitochondrial biogenesis and oxidative capacity, while reducing both central and peripheral fatigue (93, 94). This enables greater overall training loads, faster recovery, and minimizes the risk of overtraining. Adjusting training intensity to individual thresholds directly improves lactate-turnpoint velocity and running economy, thereby contributing to long-term performance gains (2). In addition, runners supplement lactate threhsold sessions in the base period with one weekly high-intensity hill repetition session (e.g., 2 × 10× 30 s at ∼6–8 mmol·L^−1^), as well as very short alactic sprints (10–15 s) (30, 31, 40).

The direct adoption of the Norwegian method has spread widely, but many athletes and coaches have also adapted its principles to their specific needs. Marathoners and middle-distance runners alike now integrate high volumes of so-called threshold intervals, and increasingly, short repetitions are also organized into sets, similar to Iglói's practice. For middle-distance runners, shorter controlled repetitions of 300–400 m at sub-threshold intensity are particularly relevant, as these velocities more closely approximate race pace while maintaining predominantly aerobic conditioning. This allows for prolonged aerobic work at speeds resembling those of middle-distance competition (41).

Such formats are conceptually linked to Iglói's use of short, controlled repetitions, which were also likely performed predominantly aerobically (78). However, the crucial difference lies in Iglói's relative neglect of longer controlled aerobic intervals (1,000–3,000 m), which in contemporary practice are seen as essential for specific endurance adaptations, such as prolonged time spent near lactate threshold, greater metabolic stability, and improved durability under sustained race-specific intensities. Meta-analyses confirm that intervals exceeding 3–5 min optimize mitochondrial biogenesis and capillary density (5, 27, 95, 96). These benefits are difficult to fully replicate using only short repetitions, even if accumulated in large volumes. Nevertheless, Iglói's genius lay in designing a system that allowed athletes to complete very high volumes of running at or near race-specific paces while maintaining relatively low internal stress. Short recoveries ensured that heart rate remained elevated, producing long periods of cardiovascular strain without reaching maximal levels, and lactate concentrations stayed moderate compared with traditional continuous threshold runs, thereby promoting lactate clearance and utilization as a fuel (33, 80). This is consistent with findings that intermittent efforts enhance monocarboxylate transporter expression and improve lactate handling (97). The alternation of different speeds and recoveries also likely recruited a wide spectrum of muscle fibers, enhancing the fatigue resistance of fast-twitch fibers in particular (98).

Moreover, while modern threshold sessions often involve repetitions lasting 45–75 s, Iglói primarily used 100–200 m repetitions (15–30 s) for similar conditioning purposes. In the literature, intervals of such short duration also exist (e.g., Billat's 30–30 or Tabata's 20–10 protocols), but these are typically performed at much higher intensities, targeting VO₂max and anaerobic pathways (15, 17, 99). Therefore, further research is needed to clarify the mechanisms and long-term effects of large-volume short-interval protocols, performed with both short and long recoveries at controlled sub-threshold intensities, in order to better understand their specific role in aerobic adaptations.

Another striking parallel between Iglói's system and modern approaches is the use of double sessions. Popularized again through the Norwegian method, double sessions allow athletes to distribute workload across two shorter bouts, which facilitates better recovery between sessions. As Talsnes et al. have demonstrated, this reduces autonomic nervous system strain and accelerates parasympathetic reactivation, thereby lowering internal load and perceived exertion (100). The resulting reduction in cumulative fatigue enhances training adherence, minimizes the risk of overtraining, and allows athletes to complete higher overall training volumes. Additionally, reduced muscle soreness and fatigue in subsequent days further support consistent performance, maximizing long-term physiological adaptations (100).

Modern comparisons highlight both strengths and limitations. On the one hand, Iglói's methods anticipated many contemporary practices, including high-frequency aerobic intervals, large sessional volumes, and even double-workout setups. On the other hand, the heavy reliance on track-based work in spikes likely imposed greater mechanical stress than is considered optimal today. Moreover, while Iglói's exclusive reliance on short intervals represents one extreme of the spectrum, current scientific understanding suggests that combining both short and long interval structures may yield the most complete endurance development.

### Lactate shuttle mechanism

A further notable aspect of Iglói's methodology is the systematic use of active recoveries, recovery sub-sets, and short “shakedown” efforts, which can be interpreted within the framework of the lactate shuttle mechanism. Lactate is not a waste product but a key energy substrate, transported via monocarboxylate transporters (MCTs) from glycolytic (type II) fibers to oxidative (type I) fibers, the heart, or liver for oxidation or gluconeogenesis. Evidence shows that 50%–75% of lactate produced during exercise is recycled, supporting metabolic flexibility, buffering capacity, and tolerance of large training volumes (101, 102). Iglói's practice of embedding short 100–150 m relaxed repetitions before and after anaerobic bouts, and inserting “recovery hundreds” between hard intervals, likely optimized this process by maintaining blood flow, sustaining oxidative engagement, and promoting faster lactate clearance (33). Mechanistically, this delayed acidosis, sustained ATP resynthesis, and allowed more high-quality anaerobic repetitions within a session, amplifying the overall adaptive stimulus.

This logic parallels modern lactate “flush” training, where active recovery at sub-threshold intensities accelerates lactate clearance compared with passive rest, with optimal removal occurring near the lactate threshold (103). In Iglói's system, jogging-based “roll-on intervals” served a similar function: keeping heart rate and oxidative metabolism engaged while lactate was recycled as fuel (43, 104). This not only improved lactate kinetics but also increased mitochondrial density and oxidative enzyme activity, supporting higher workloads without premature fatigue. Importantly, such recoveries ensured athletes remained physiologically engaged even during rest periods, thereby maximizing session efficiency.

Another innovation was his use of relaxed but relatively fast 100 m runs at the end of sessions under accumulated fatigue. These cooldown efforts likely promoted additional motor unit recruitment via Henneman's size principle, as fatigued slow-twitch fibers necessitate fast-twitch engagement to sustain force output. This extended the conditioning stimulus, improving firing rates, synchronization, and neuromuscular economy (105, 106). Comparable to Snell and Lydiard's late-fiber recruitment during long runs, these efforts also had psychological benefits, reinforcing technical precision and relaxation under fatigue, conditions most similar to the decisive phases of racing (107).

The integration of these strategies illustrates how Iglói's approach anticipated modern endurance training principles. For example, Canova's alternation workouts, where threshold and race-pace efforts are interspersed, reflect the idea that “the rest is the work” (108). Similarly, lactate “flush” repetitions in running or cooldown rides in cycling apply the same principle of sustaining metabolic engagement during recovery (109). These practices improve lactate transport capacity, oxidative metabolism, and buffering systems, delaying acidosis and extending time to exhaustion (110).

Today, these same principles are can be actively reintroduced in anaerobic, race-specific training, where structured active recoveries and recovery subsets allow athletes to perform significantly greater volumes of high-quality work within a session, creating an additional and powerful training stimulus that enhances adaptation beyond what traditional interval formats can provide.

### Running economy

One of the most persistent misunderstandings of Iglói's methodology is that his intervals were primarily time-based. In reality, most were effort-based, with athletes focusing on rhythm, relaxation, and smooth mechanics rather than strict split times (75, 76). Iglói often corrected posture and relaxation during sessions, adjusting workouts based on quality (56). This approach likely trained running economy, the oxygen or energy required to sustain a submaximal running speed, directly at race paces, embedding efficiency into the neuromuscular system under real competitive intensities. Running economy is a core focus in endurance research because it predicts performance in distance events, often differentiating athletes with similar VO2max (111, 112). Improvements, typically 2%–5%, arise from better biomechanics, muscle elasticity, and neural efficiency, allowing faster times without boosting aerobic capacity. Studies explore it to identify trainable factors like stride mechanics or strength work, guiding coaches to enhance endurance while avoiding overtraining (112, 113)

Effort-based intervals served dual purposes: managing physiological stress and reinforcing biomechanical precision. Iglói introduced “swings” as deliberate stride variations for versatility, short swings boosted frequency with shorter steps, while long swings emphasized length and floating motion (66, 76). Practicing both created multiple “gears,” helping athletes shift muscle recruitment, delay fatigue, and adapt tactically in races. Key distinctions include mechanical efficiency (smooth movement and force transfer) vs. metabolic efficiency (sustainable energy use); they interconnect, but stride variability training lowers energy costs across paces (114). Iglói harmonized them via high-repetition, relaxed practice, prioritizing smooth running over forced effort.

This flexibility shines in races: a top 10,000 m runner used long swings (low frequency, extended length) most of the way, then switched to high-frequency short swings for a winning kick, while rivals struggled with rigid styles. Stride-length runners need strength focus; frequency-based ones benefit from turnover drills. Versatile mechanics under fatigue improve late-race efficiency (115). Overall, Iglói's model integrates physiology and biomechanics: effort-based intervals, relaxation, and swings built race-specific economy—not just lower oxygen use, but sustained efficiency with tactical adaptability.

### Periodization

Iglói developed the core of his training methodology well before the formalization of linear periodization, predating the popularization of mesocycles and the hard-easy approach by figures such as Matveev, Lydiard, and Bowerman (18, 116). Although his system incorporated timing elements to optimize performance, it eschewed rigid mesocycles, leading contemporaries to critique its ability to reliably peak athletes; critics often pointed to underperformances at events like the 1954 European Championships in Bern and the 1956 Olympics as evidence of this limitation. However, subsequent results demonstrated that many of Iglói's athletes achieved peak performances in major competitions, largely because he scheduled races based on observed readiness rather than predetermined dates. Despite the appearance of uniform daily training, closer analysis reveals a discernible weekly pattern with alternating higher- and lower-intensity days, facilitating optimal workload distribution and recovery. Periodization research supports this flexibility, indicating that undulating models enhance physiological adaptations by varying stimuli to prevent training stagnation and overreaching. Furthermore, such non-linear approaches allow for individualized adjustments, promoting greater long-term improvements in endurance capacity through autoregulated intensity variations (117).

A striking difference in Iglói's system is that he structured lighter-load days using intervals, whereas modern training paradigms almost exclusively employ low-intensity continuous running for recovery (118). This interval-based approach on easy days holds relevance from multiple perspectives, as it maintains mechanical loading on the muscles while moderating overall intensity, potentially fostering adaptations such as improved neuromuscular efficiency and tendon resilience without excessive metabolic strain (85, 118). In contrast, exclusive reliance on low-intensity runs may limit these benefits, though it excels in enhancing mitochondrial biogenesis and capillary density for aerobic base building.

Another parallel with contemporary methods is Iglói's integration of mixed intensities within microcycles throughout the year, with progressive manipulation across the season (41). Rather than confining low-intensity work to early preparation phases and high-intensity efforts to later ones, these elements were consistently present in every microcycle, albeit in varying proportions through interval adjustments. This mirrors modern periodization, which often features pyramid-like distributions that increasingly polarize as the season advances, ensuring all training zones remain active to sustain metabolic flexibility and prevent detraining (18, 85, 119, 120). Such concurrent training enhances overall performance by optimizing the interplay between aerobic and anaerobic systems, as evidenced by improved lactate threshold and VO₂max in polarized models (121, 122).

Iglói also pioneered strategic warm-ups and race priming, where athletes performed harder strides or brief intervals the day before competitions to elevate muscle tension, prime VO₂ kinetics, and engage the aerobic system prior to racing (123, 124). Psychologically, Iglói fostered resilience through unpredictable session structures, unknown splits, workouts, and sets, training athletes to self-regulate effort without relying on timers. Additionally, the use of watches, tests, and time trials improved athletes' mental fortitude, teaching them not to fear pain or competition, thereby building psychological endurance essential for high-stakes events (125, 126).

### Strenghts and limitations

A major strength of my study is that, to my knowledge, I am the first to systematically reconstruct and present Mihály Iglói's life and coaching methodology with a scientific approach. I achieved this using bilingual sources in both Hungarian and English, reflecting Hungary and the United States, the two countries where he worked the most and achieved his greatest results. In addition to the literature, I incorporated feedback from athletes who were directly coached by Iglói, ensuring authenticity. Further strengths include situating his work within its historical context and the novel approach of comparing his methods with contemporary, peer-reviewed scientific studies, highlighting potential applications and adaptations for modern practice.

Nevertheless, several limitations must be acknowledged. The narrative nature of the study inherently introduces the risk of my interpretation and bias. Historical documentation is inevitably incomplete, and personal recollections are selective and may lack comprehensiveness. Moreover, Iglói's methodology was highly individualized and evolved over decades. Comparative analysis with modern methods may be affected by differences in the scientific approaches of their respective eras, as well as terminology, introducing potential interpretational or translation errors. Additional limitations include potential language-based challenges when synthesizing bilingual sources, and the fact that certain aspects of Iglói's training practices were undocumented or codified, preventing full verification. Furthermore, the reliability of non-peer-reviewed sources is often questionable, as these frequently provide only fragmented accounts rather than complete descriptions of his methods.

## Conclusion and practical applications

In conclusion, this study reconstructed Mihály Iglói's innovative interval-based training system through historical analysis and comparative review, highlighting its mid-20th-century roots and pioneering features that anticipated modern endurance concepts. His contributions included the systematic use of sets, effort-based intervals, differentiated running techniques, and complex manipulation of workloads for distinct physiological and tactical purposes. His professional rigor and motivational ability created a methodology that produced extraordinary performances across different countries and generations. Such historical analyses preserve coaching heritage and generate insights that can be translated into contemporary practice to advance endurance training.

From a modern perspective, full reapplication of Iglói's system is not recommended; instead, a retooling approach is advised, adapting its mechanisms to complement contemporary, evidence-based and sport science–supported practices. Short intervals performed in sets at near-competition speeds with brief recoveries can enhance aerobic conditioning and prepare athletes for repeated high-intensity efforts. Proper manipulation of these intervals emphasizes efficient running mechanics, while their differentiated application across tactical situations, such as pacing, surges, or finishing fosters versatility and race-specific readiness. Incorporating varied sets, active recovery to support lactate shuttle, and “flush” intervals promotes improved lactate utilization and sustained work volume. Prioritizing effort-based intervals over strict timing allows athletes to self-regulate intensity according to perceived readiness. These principles underscore the practical applications of Iglói's system in modern endurance training, bridging historical insight and contemporary implementation.

## Data Availability

The original contributions presented in the study are included in the article/Supplementary Material, further inquiries can be directed to the corresponding author.
